# Global Frameworks Toward Effective Integration of Technology in National Dementia Strategies: Narrative Review and Case Study in Mexico

**DOI:** 10.2196/83861

**Published:** 2026-10-22

**Authors:** Lili Marlene Camacho-Bustamante, Claudia I Astudillo-García, Guillermo Velázquez López, Luis Gerardo Moncayo-Samperio, Alberto Agustín Palacios-García, Sergio Alberto Navarro-Tuch

**Affiliations:** 1 Department of Mechatronics, School of Engineering and Sciences, Tecnologico de Monterrey, Campus Ciudad de México, Prol. Canal de Miramontes, Coapa, Mexico City, 14380, Mexico, 52 555 483 2025; 2 Dirección de Investigaciones Epidemiológicas y Psicosociales, Instituto Nacional de Psiquiatría Ramón de la Fuente Muñiz, Mexico City, Mexico; 3 Dirección de Servicios Clínicos, Instituto Nacional de Psiquiatría Ramón de la Fuente Muñiz, Mexico City, Mexico; 4 Department of Medical Sciences, School of Medicine and Health Sciences, Tecnologico de Monterrey, Campus Ciudad de México, Mexico City, Mexico

**Keywords:** dementia, technologies, policy analysis, literature review, health policy

## Abstract

**Background:**

Population aging is a global phenomenon that has led to a rise in age-related conditions, particularly dementia. This situation presents significant challenges for health care systems, caregivers, and society as a whole. Technologies have shown potential to address the needs of people living with dementia and their caregivers, offering support in daily activities, communication, and care monitoring. However, only a quarter of countries worldwide have developed national dementia plans, and even fewer have integrated technology-based solutions into these policies.

**Objective:**

This study aimed to analyze existing gaps in dementia plans, with a particular emphasis on the limited integration of technological solutions as essential tools for dementia care and management. Based on this analysis, this study proposes a framework to facilitate context-appropriate technology integration.

**Methods:**

This study presents a narrative review of public policy frameworks for dementia care. The review provides a global overview, examines the context in Latin America, and focuses on Mexico as a case study to analyze how technology is integrated into dementia strategies.

**Results:**

Based on these findings, this study proposes a framework of general recommendations adaptable across multiple countries. Furthermore, it demonstrates the application of this framework in Mexico to strengthen its national dementia strategy through technological solutions tailored to people living with dementia and their informal caregivers.

**Conclusions:**

Given that dementia is recognized as a global priority, countries with aging populations have developed numerous technological solutions. These international experiences serve as valuable references to guide the adoption or rejection of specific approaches, provided that local cultural and social contexts are considered. The applied technologies were merged into a framework that can be adapted to different countries, taking into account social aspects, governance, and region-specific barriers to generate a viable, customized option, as demonstrated in the case study focused on Mexico.

## Introduction

Dementia poses a major challenge for all countries. However, it cannot be addressed without first considering the broader context of aging. Aging is defined by the World Health Organization (WHO) as the gradual accumulation of molecular and cellular damage [1]. These modifications are natural, unavoidable, and start in early adulthood, generating physical and mental changes [2]. In addition to biological changes, aging often entails significant life variations, such as retirement or the loss of close friends and partners [3].

Projections estimate that by 2050, one in 5 individuals worldwide will be aged 60 years or older, totaling between 1.6 and 2.1 billion older adults [4,5]. Aging also increases the risk of several chronic disorders like neurodegenerative, cardiovascular, and metabolic diseases, as well as other disorders [6].

Among these conditions, dementia is particularly significant. Dementia is a syndrome that may be caused by several diseases and causes cognitive decline [7]. The most common types of dementia include Alzheimer disease (AD), which accounts for approximately 60%-80% of cases, followed by vascular dementia (5%-10%), dementia with Lewy bodies (around 5%), and frontotemporal dementia (approximately 3%) [8]. The most common symptoms of this cognitive impairment may include memory loss, changes in behavior, issues with visuospatial perception, and language problems such as difficulty following conversations [9]. Dementia also impairs the ability to perform daily activities and eventually leads to a loss of individual autonomy [10].

Global costs associated with dementia are typically classified into 3 main categories, including direct medical costs (formal health care services), direct social costs (formal care outside hospital settings), and indirect costs (informal care provided by family members) [11,12].

Because of the importance of this issue and its high cost for governments around the world, as well as the growing number of people affected with between 152 million cases of dementia by 2050 [13], many countries have created public policies to address dementia. These policies aim to meet the needs of people living with dementia and their caregivers.

To effectively address the rising rates of dementia, public policies and action plans must adopt a holistic approach that accounts for the diverse causes and contributing factors of these conditions. In this context, the integration of technology into public policy represents a promising strategy to transform dementia care [14].

The integration of technology into dementia care is increasingly recognized as a key element to enhance early detection, optimize patient management, and improve caregivers’ support. Advances in digital health tools, AI, and remote monitoring systems offer promising solutions to address the complex needs of people living with dementia. These technologies can bridge gaps in access to specialized services, particularly in underserved regions, and contribute to more personalized and effective interventions.

One approach to support people living with dementia is the use of assistive technology (AT), along with other technologies for early detection and prediagnosis. AT refers to any devices, tools, or systems that help improve or maintain a person’s functional abilities [15,16]. Table 1 shows the types of devices that have been developed based on their specific functions.

**Table 1.** Types of assistive technologies. Authors’ own creation using data adapted from Klimova et al [16].

Classification	Description
Devices for daily living	Technologies that provide aid with memory, orientation, communication, and other daily activities.
Safety devices	These devices aim to safeguard individuals in cases of disorientation or wandering, and to assist in the prevention of associated safety risks.
Telecare devices	These devices offer services and enable access to support systems, facilitating continuous monitoring and providing assistance when necessary.
Devices to support engagement, social participation, and leisure	Technologies that enhance quality of life by facilitating social interaction and engagement.

This study aimed to evaluate current dementia-related policies from a technological perspective and to propose strategies for the effective integration of technology into dementia care. The analysis progresses from global policies to more specific national policies. It culminates in a Mexican case study that explores potential approaches for integrating technology into national dementia policies.

The first section of this research presents a summary of various global policies and strategies, highlighting their evolution and the extent to which they incorporate technological solutions to address key challenges. Subsequently, an overview of dementia plans in selected Latin American countries and Mexico is provided, as Mexico will serve as the case study in subsequent sections. Thereafter, a framework for technology implementation is presented, taking into account the social context, governance, and regional public policies. Finally, the case study of Mexico is presented to demonstrate the application of this approach.

The main contributions of this paper are as follows:

- Policy-oriented review: a specialized review focused on the integration of emerging technologies within existing public dementia policies.
- Technology identification: identification of current technological tools and their formal inclusion in public health frameworks.
- Adaptive implementation framework: an adaptable decision-making framework designed to bridge the gap between public policies and technological implementation. While Mexico is presented as an illustrative case study to demonstrate its practical application, the framework is fully transferable to other national contexts.

## Methods

### Objective

The objective of this narrative review was to analyze the global landscape of public policies on dementia, with a specific focus on the application of technological solutions and an emphasis on Mexico, which is subsequently analyzed as a case study. This analysis was conducted from the perspective of technological implementation, evaluating how official policy frameworks incorporated digital tools and innovation to address the dementia crisis at global, regional, and national levels.

### Search Strategy

This review aimed to examine the evolution and current state of the field from the perspective of key stakeholders. Consequently, global analysis incorporated reports and guidelines from the WHO, Alzheimer’s Disease International (ADI), and Pan American Health Organization (PAHO), given the review’s focus on Latin America.

The search strategy used specific terminology across various databases. For WHO and ADI, the following Boolean query was applied: (public policy OR guide OR plan) AND dementia AND (technology OR tech OR digital OR assistive). The PAHO search was limited to (dementia AND policy) because the platform offered limited search customization. Furthermore, the search was supplemented by a direct review of regulatory documents to analyze the evolution of public policies on dementia, tracing their transition from a general aging context to their current global importance.

Regarding stakeholder websites, publications intended for general awareness or expressing opinions about dementia were excluded. Only reports and guidelines addressing global initiatives focusing on Latin America or discussing the use of technology in dementia care were considered. A screening process was conducted for studies that passed the initial filter, and the relevance of each was discussed with a supervisor. The use of gray literature was selected because it contains the documents related to public policies from different countries. However, the search was limited to the official websites of the main organizations involved in developing or promoting general public policies and dementia-specific policies.

Additional reports referenced within the studies identified during the initial search were also included in order to complement the review.

### Data Extraction and Classification

Data extraction was focused on authorship, publication year, primary themes, and document type. For reports, all stakeholders involved in the drafting process were identified. The classification of entries was primarily divided into 3 categories, namely reports, news, and hubs. The latter category served to group related items of the same nature, such as entries that aggregated all dementia-related report results within a specific time period.

Related hubs and news sources were reviewed, and those that did not contain official documents were excluded to retain only official reports.

## Results

### Overview

The initial search of the WHO website yielded 52 results. Of these, 16 met one of the inclusion criteria, specifically addressing global public policies, focusing on the designated area of interest, or discussing the implementation of technology in dementia care. From these, only 3 official reports were ultimately included.

The search conducted in the ADI database yielded a total of 486 results. However, the most were excluded because they were not formal reports or focused on geographical regions outside the scope of this study.

Consequently, 65 results were selected, of which 25 were reports, primarily consisting of editions of the World Alzheimer Report and supporting documents from From Plan to Impact. Annual follow-up reports on the status of dementia policies were also identified.

The PAHO website search yielded 28 results, of which only one was considered relevant and classified as a technical document. Additionally, other reports cited within the analyzed literature were included. These documents were deemed essential for the development of this review to ensure an exhaustive approach and to provide a comprehensive overview of the current state of public policies on dementia. The review identified key reports with global, Latin American, and Mexican relevance; selected reports are presented in Table 2.

**Table 2.** Summary of the main reports identified. Authors’ own creation using data adapted from [5,11,17-34].

Title and year	Issuing organization	Geographic scope	Core focus
Global status report on the public health response to dementia (2021) [19]	WHO^a^	Global	Evaluates progress on multisectoral efforts to address dementia globally; identifies implementation barriers of the global action plan and updates dementia burden and cost estimates
Decade of healthy aging: plan of action (2020) [5]	WHO	Global	Ten-year collaborative plan to improve lives of older adults, their families, and communities; placing older adults at the center
Global action plan on the public health response to dementia 2017-2025 (2017) [17]	WHO	Global	Seven priority areas of action to improve the lives of people with dementia and caregivers; include prevention, diagnosis, treatment, care, and caregiver support
The global impact of dementia 2013‐2050 (2017) [20]	ADI^b^	Global	Policy brief updating prevalence figures, from 44 million in 2013 to 135 million by 2050; 71% will live in low- and middle-income countries by 2050.
Dementia: a public health priority (2012) [18]	WHO and ADI	Global	Joint report published in 2012 that raised awareness of dementia as a public health priority and addressed its global impact, costs, and the need for national and international action
World Alzheimer reports [11,26-34]	ADI	Global	Annual series of comprehensive reports on the global socioeconomic impact of dementia; each report addresses a different topic (2024: attitudes and stigma; 2025: rehabilitation; 2023: risk reduction; 2022: postdiagnostic support; 2021: diagnosis).
From plan to impact reports [21-25]	ADI	Global	Annual series monitoring member states’ progress toward the WHO Global Action Plan; 7 chapters cover the 7 action areas of the plan

a WHO: World Health Organization.

b ADI: Alzheimer’s Disease International.

### Global Dementia Policies and Technology Integration

To propose a comprehensive approach to technology integration, it is necessary to understand the current state of global strategies and policies that prioritize dementia and the aging population. This understanding helps identify gaps and guide future policy development to better address the needs of individuals living with dementia and older adults.

The significance of this issue is underscored by the efforts of various organizations. In response to the global aging population and its resulting consequences, organizations such as the Alzheimer’s Association, WHO, and the United Nations have emphasized the inclusion of this topic on the political agenda. They advocate developing strategies aimed at improving the quality of life for people living with dementia and their caregivers. However, according to the WHO, only a quarter of all countries have implemented a national plan or strategy to address dementia for 2021 [14]. Half of these countries are in Europe, and many of their plans had expired or were nearing expiration.

This global response is further illustrated in Figure 1, which demonstrates the chronological evolution of dementia policies and the primary events that have made dementia a visible and essential issue on the global stage. Because most people living with dementia are aged 60 years or older, early initiatives primarily focused on the needs of the older adults.

**Figure 1.**
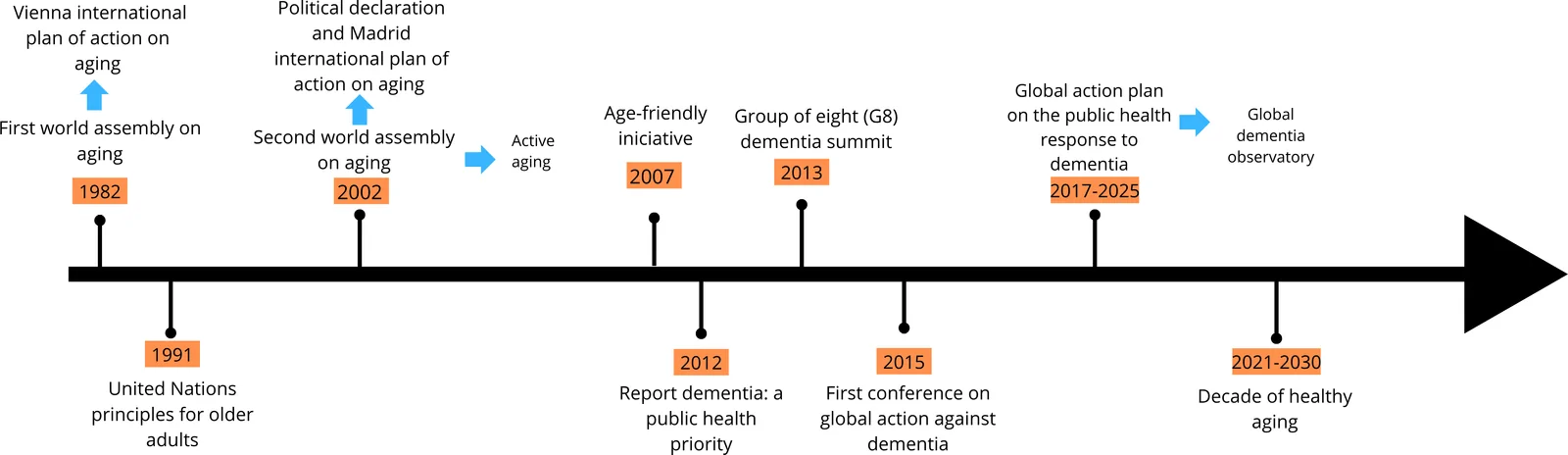
Evolution of initiatives and strategies about dementia. Authors’ own creation using data adapted from [5,17,18,35-38].

The initial collaborative action addressing dementia was the First World Assembly on Aging in Vienna in 1982 [39]. This early initiative emphasized the imminent aging of the global population and focused on addressing humanitarian and developmental issues, including the needs of older adults and their role within society. As a result of this meeting, the International Plan of Action on Aging was developed. The first Action Plan on Aging sought to increase awareness of the challenges that population aging would present across all sectors. Its objectives included raising awareness of the economic, social, cultural, humanitarian, and socioeconomic implications of population aging [40].

Despite its intentions, the recommendations outlined in the first Action Plan on Aging lacked specificity regarding the actions needed to achieve its objectives. The plan also lacked specific timelines and measurable goals. Nevertheless, it served as a foundational reference for the development of more robust initiatives.

In response to the growing importance of aging, the United Nations established the Principles for Older Persons in 1991 [37]. The 18 principles address 5 areas: independence, participation, care, self-fulfillment, and dignity. By 2002, the primary focus remained on aging as a broad social issue. In response, the Second World Assembly on Aging was held in Madrid, resulting in the adoption of the International Plan of Action on Aging. This political declaration, composed of 19 articles, emphasized the need to incorporate aging into public policy. Its main priorities included older adults and development, promoting health and well-being in old age, and creating enabling and supportive environments [35]. Notably, the document emphasizes the role of technology as a critical tool in addressing the challenges associated with aging.

In 2012, the Global Report on Dementia was published, calling for urgent action on dementia worldwide [18]. Regarding technology, the report discussed ethical issues such as privacy and security, described existing tools, and highlighted the need for more research and evaluation to show the benefits of technology in dementia care.

In 2013, the G8, a group composed of the industrialized countries and representing approximately half of the world’s economic activity, held the Dementia Summit in London [38]. At this event, ministers, researchers, and pharmaceutical companies gathered to discuss the importance of addressing dementia and the major challenges it presents, such as the lack of funding for dementia research.

In 2015, the first Conference on Global Action Against Dementia was held. The goal of this conference was to outline future action plans and to strengthen the call for all countries to recognize dementia as a public health priority. The conference also highlighted the importance of developing technological innovations to support people living with dementia, as well as the need for more information on how to effectively integrate these technologies into dementia care. It also stressed that national dementia policies must include a defined role for technology within coordinated care strategies [36].

These collaborative efforts culminated in the presentation of the Global Action Plan on the Public Health Response to Dementia in 2017 [17]. This plan specified objectives to be achieved during the period of 2017‐2025 and the elements to be evaluated. In this document, technology was mentioned mainly in the seventh strategy action area, dementia research and innovation. The proposal included increasing investment in research focused on technological innovation in health care, particularly in the development of tools to address the needs of people with dementia and their caregivers. It also emphasized the importance of providing access to assistive technologies and encouraging the active participation of individuals living with dementia in the evaluation and design of these solutions.

To monitor progress on the goals of the Global Action Plan, the Global Dementia Observatory (GDO) was created [41]. It defines specific requirements for each area of action. In relation to technology use in care, only one of the ten indicators includes a subindicator that evaluates the availability of care products and assistive technologies. This subindicator considers 3 categories, including adult hygiene products, home modifications, and assistive devices. However, it only includes basic tools such as walking frames and hearing aids, without explicitly mentioning more advanced technologies like tracking systems or ambient assisted living.

Although some health policies address the use of AI and its ethical and legal implications, they often do so from a general perspective, without focusing specifically on dementia. For example, the WHO’s Ethics and Governance of Artificial Intelligence for Health (2021) [42] acknowledges the role of digital tools and innovation but offers no specific guidance on their development, implementation, or evaluation in dementia care. This lack of direction limits the effectiveness of current policies and highlights the need for frameworks that clearly define the role of technology in improving outcomes for people living with dementia.

As a significant milestone, The Decade of Healthy Aging (2021‐2030) [5] is a 10-year initiative aimed at changing perceptions about aging, promoting age-friendly environments, creating integrated and responsive health care systems, and ensuring access to long-term care for older adults. The initiative also emphasizes the role of technology in enhancing care for older adults. Its principal contributions to the technological framework encompass the development of standards for access to information and communication technologies and assistive devices, the promotion of affordable and effective digital tools, and the implementation of innovative long-term care services.

### Latin American Dementia Policies: Regional Technology Landscape

Population aging is a phenomenon affecting the entire world, including Latin America, leading to a significant increase in dementia cases. In fact, in Latin America, dementia is the sixth leading cause of death and the primary cause of dependency [43]. Additionally, the prevalence of dementia in the region is 8.5% among individuals aged 60 years and older, the highest in the world; this figure is expected to reach 19.3% by 2050 [44].

The lack of formal studies in various Latin American countries makes it difficult to understand the existing issues and forces the generalization of incidence, prevalence, and cost figures, relying on countries that have conducted more studies, such as Cuba, the Dominican Republic, Mexico, Peru, and Venezuela [43].

The development and evolution of policies and plans to combat dementia have become crucial as countries acknowledge the growing proportion of their populations aged 60 years and older. This demographic shift emphasizes the prevalence of common illnesses and conditions associated with aging. Nations have adopted 2 main approaches: incorporating dementia initiatives within broader national plans that also address other neurodegenerative diseases or developing specific strategies targeting dementia or its most common type, AD. For instance, Mexico’s initial plan focused on AD, while countries like Chile and the Dominican Republic have implemented general plans targeting dementia [45].

According to ADI, as of 2024, 8 countries in Latin America (Brazil, Chile, Cuba, Costa Rica, Dominican Republic, Mexico, Puerto Rico, and Uruguay) had a formal dementia plan, with 2 more countries (Panama and Peru) developing their plans. A review of policies from 6 Latin American countries indicates that all these plans align with the recommendations and action areas proposed by the WHO in the Global Action Plan on the Public Health Response to Dementia [17]. These areas include early diagnosis, staff training, public awareness campaigns, and improving the quality of life for people living with dementia. Additionally, each plan includes objectives tailored to their specific context and needs, such as enhancing monitoring and data collection related to the disease [45-49]. All these plans are acknowledged by ADI as the most recent for each country (Multimedia Appendix 1).

Some of these plans were established many years ago and have yet to be updated, such as those of Cuba and Chile [46,48]. Others were designed with a long-term perspective, like Costa Rica’s plan, which was set to last 10 years until its deadline in 2024 [50]. Currently, many of these plans are undergoing updates. For example, Mexico’s dementia plan, initially created in 2014, was updated in 2024.

In these plans, early diagnosis is prioritized through the training of frontline staff and the use of existing resources, as opposed to creating new services. Another important aspect is the inclusion of action lines aimed at informing people living with dementia about their legal rights.

Most of the dementia plans and strategies reviewed mention technology only as a tool for sharing information with caregivers and people living with dementia. Some also refer to promoting innovation in research. However, only Chile’s plan explicitly emphasizes the use of technology to improve quality of life and the development of devices to support people with dementia [46].

### Mexico’s National Dementia Strategy

#### Overview

Dementia represents a growing concern in Mexico. Currently, an estimated 1.3 million people in Mexico are living with dementia, and projections indicate that this number could increase to 3.5 million by 2050 [47].

In response to this challenge, the first national plan specifically addressing dementia was introduced in 2014 under the title “Plan de Acción Alzheimer y Otras Demencias.” This initiative aimed to enhance the well-being of individuals living with dementia by reinforcing the health care system through 7 key objectives. These included eliminating stigma, improving access to accurate information for patients and caregivers, and developing a comprehensive care system that integrates medical, social, and community support.

Despite the plan’s commitment to periodic monitoring and evaluation, a formal update was not introduced until 2024 with the publication of the National Dementia Plan [47]. While this new strategy retains core objectives, such as eliminating stigma and supporting scientific research, it introduces additional measures aimed at strengthening early detection through a comprehensive care model. The primary goal of these enhancements is to improve patient outcomes and reduce the prevalence of dementia-related complications. A key addition to this plan is the strategic involvement of civil associations, which play a critical role in raising awareness and disseminating information about dementia. The plan also prioritizes the reinforcement of the primary health care system by integrating systematic screening processes and equipping health care professionals with improved diagnostic tools and training. These initiatives seek to establish a more effective and accessible support system for individuals living with dementia and their caregivers.

As observed, the application and development of technologies are largely absent from both new and existing plans. Therefore, it is essential to identify the key sectors where the use and development of technological solutions can be effectively integrated and leveraged for their potential impact.

The following points summarize the most pressing needs that must be addressed to build an effective and inclusive dementia care strategy in Mexico related to the application of technologies:

#### Strengthening Primary Health Care Networks

Primary health care providers, including those within the Mexican Social Security Institute (Instituto Mexicano del Seguro Social [IMSS]) and the Institute of Social Security and Services for State Workers (Instituto de Seguridad y Servicios Sociales de los Trabajadores del Estado [ISSSTE]), should receive training to recognize early signs of dementia, ensure timely referrals to specialized care, and use appropriate technologies for screening and early detection.

#### Support for Informal Caregivers

Given the cultural significance of family caregiving in Mexico, it is essential to implement policies that provide effective support to informal caregivers. This support may include financial assistance, respite care services, and access to training programs delivered through the Secretariat of Welfare or local community centers to strengthen caregiving capacity and enhance the quality of care provided.

Within this framework, the Mexican government has introduced the Salud Casa por Casa program, which delivers home-based health care services to individuals aged 65 years and older [51]. The program is structured across 4 levels of care that respond to varying degrees of clinical need, encompassing stable conditions as well as complex cases that require continuous medical attention and sustained caregiver involvement.

This initiative presents a valuable opportunity to integrate smart environments into long-term care and advance the development of large-scale technological solutions aimed at monitoring individuals with dementia. Such innovations can support early detection, ongoing cognitive assessment, and personalized care interventions within the home setting. However, this program remains in the process of structuring and implementation, and it is not yet operational.

#### Public Awareness and Education Campaigns

A nationwide public awareness campaign should be launched to reduce the stigma associated with dementia. The campaign could be spearheaded by the Ministry of Health, in collaboration with nongovernmental organizations (NGOs) like Federación Mexicana de Alzheimer (FEDMA), to educate the public about the importance of early diagnosis and the available resources. Notably, efforts are already underway through the Dementia Friends initiative, introduced in Mexico by FEDMA in 2018, with the primary objective of eliminating stigma [52].

#### Investment in Research and Innovation

To address the growing challenge of dementia in Mexico, it is crucial to increase funding for research through institutions like the Secretariat of Science, Humanities, Technology, and Innovation (SECIHTI). This funding should focus on studies investigating genetic and environmental factors contributing to dementia within the Mexican population and developing culturally appropriate care models. Using databases with cohorts on the health of the Mexican population offers significant opportunities to analyze data for better policies and informed decisions.

Since 2001, the Mexican Health and Aging Study (MHAS) has gathered essential data for policy development and health care strategies. Based on a nationally representative sample of 15,186 Mexicans aged 50 years and older, this longitudinal study collects information on health status, socioeconomic conditions, family structure, living environments, psychosocial factors, and major life events [53]. MHAS has expanded to include specialized assessments like cognitive evaluations and biological sample collection, providing valuable insights into aging and dementia.

Additional cohorts, such as the Cohorte de Obesidad, Sarcopenia y Fragilidad de Adultos Mayores Mexicanos, offer longitudinal data on sociodemographic information, cognition, nutrition, and other health aspects [54]. The Encuesta Nacional de Salud y Nutrición (ENSANUT) provides data on risk factors like smoking, diabetes, and cardiovascular disease [55]. Integrating these rich data sources can help Mexico advance research, develop effective policies, and create culturally tailored interventions to reduce the impact of dementia and other health challenges.

#### International Collaboration

Mexico should actively engage in international forums and collaborative initiatives focused on dementia care. This participation could involve partnerships with organizations such as the WHO and the PAHO, as well as the establishment of bilateral agreements with countries that have implemented advanced dementia care strategies. Through these collaborations, Mexico could both benefit from and contribute to the exchange of innovative technologies and best practices, particularly in the areas of early diagnosis and nonpharmacological treatment approaches.

#### Implementation of Community-Based Care Models

Community-based care models, such as those implemented by the Network of Municipalities for Health (Red de Municipios por la Salud) [56], should be expanded to include dementia-specific services. These models could provide localized support and resources for individuals living with dementia and their families. Additionally, they could facilitate the formation of dedicated teams to assess the acceptability, usability, and effectiveness of technological devices designed for people with dementia and their caregivers, thereby promoting more responsive and context-appropriate innovations in care.

### Technology Implementation Framework

Technology implementation aligned with global and national strategic goals requires a structured procedure. This procedure considers overarching global expectations before evaluating country-specific conditions. This approach is presented first in general terms and is subsequently illustrated through a case study focused on Mexico. The objective of this framework is to allow adaptation to the specific characteristics of different national contexts.

This section presents a framework, as depicted in Figure 2, which first outlines the global landscape with its corresponding policies, strategies, and the technologies to be implemented within each key area. Subsequently, a case study focused on Mexico is presented to illustrate the application of this framework in a specific environment. The intersection of regional policies, governance, and the social and cultural context is evaluated to identify constraints and barriers, thereby determining the viable options for this country.

**Figure 2.**
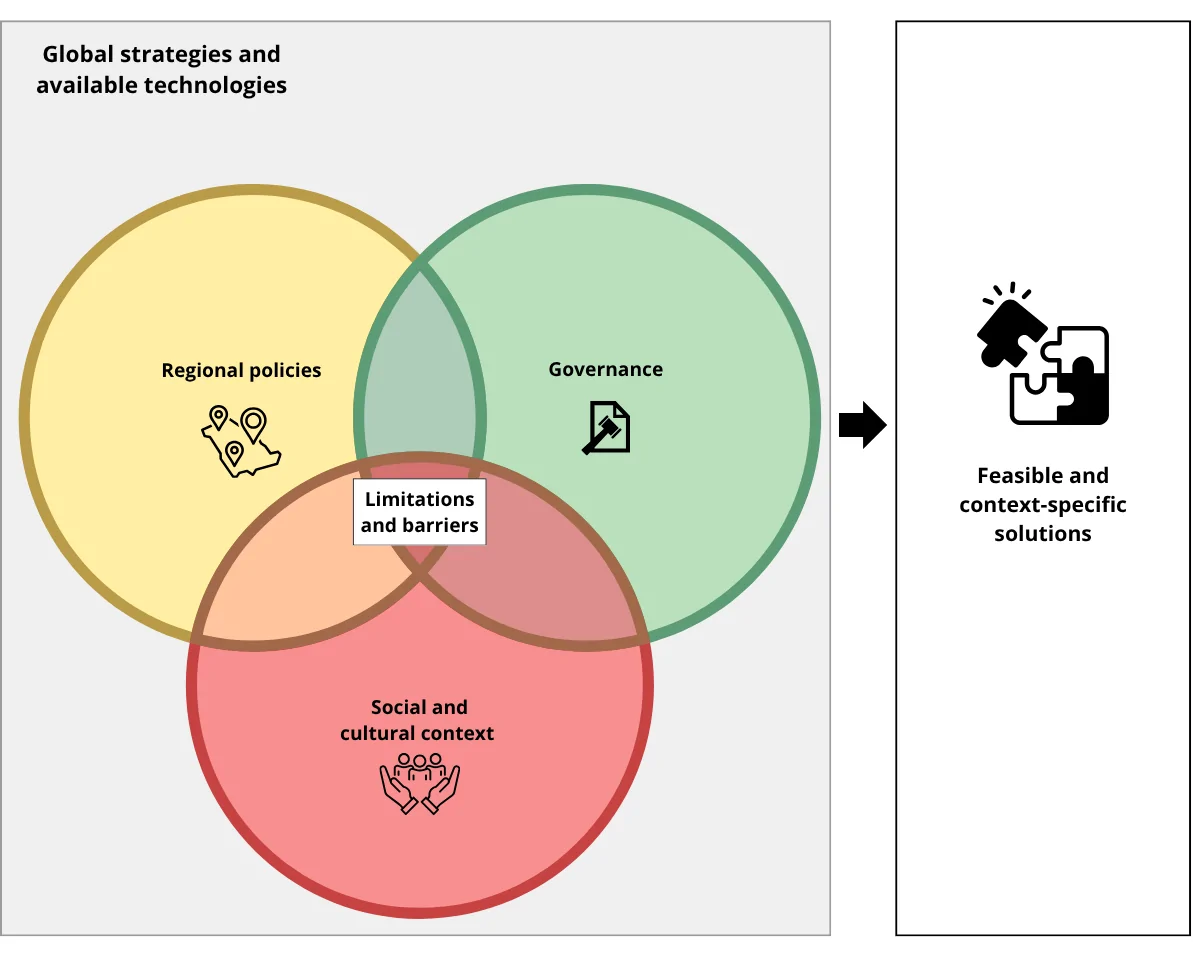
Conceptual framework for contextualizing global technology recommendations.

Following the analysis in the previous sections, the main areas where various policies converge were identified. Consequently, the action areas specified in the Global Action Plan on the Public Health Response to Dementia were selected as the foundational baseline, as shown in Figure 3. These areas serve to identify core priority approaches and determine which existing technologies can facilitate the fulfillment of their objectives.

**Figure 3.**
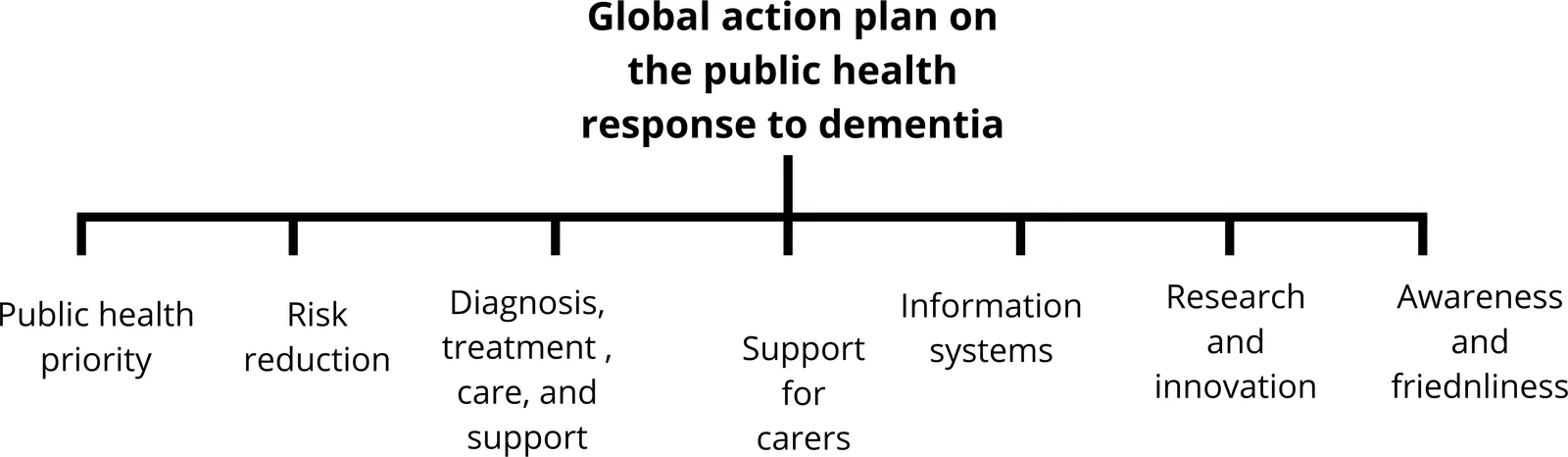
Key strategic areas of the World Health Organization (WHO) global action plan on dementia. Authors’ own creation using data adapted from [17].

Based on the analysis, the main issues addressed in global plans and proposals were identified and summarized in Table 3. Along with each specific strategy, the table outlines key problems within each domain, the technologies previously applied to address these issues, and specific examples from the literature. A more detailed examination of these applications follows.

**Table 3.** Technologies implemented based on the intervention strategy.

Strategy, issue, and technologies	Approach
Diagnosis, treatment, care, and support	
Underdiagnosis	
Machine learning	[57-62]
Data collection and tracking	[63,64]
Virtual reality	[65,66]
Automatized screening tools; apps for tablets and smartphones	[67,68]
Training	
Virtual reality	[69,70]
BPSD^a^	
Virtual reality	[71,72]
Cognitive training and rehabilitation	
Virtual reality	[69]
Support for carers	
Social interaction	
Virtual reality, wearables, social robots, and generative AI	[73,74]
Long-term care	
Smart environments and wearables	[75-77]
Safety and monitoring	
Wearables	[78,79]
Managing care	
Generative AI	[80]
Awareness and friendliness	
Removing stigma	
Virtual reality	[81]
Awareness	
App for tablets and smartphones	[82]
Risk reduction	
Risk index	
Machine learning	[83,84]
Cognitive training	
Virtual reality, apps for tablets and smartphones, and video games	[85-87]
Information systems	
Cohort construction	
Electronic health records	[88,89]

a BPSD: behavioral and psychological symptoms of dementia.

Integrating technology into dementia care improves effectiveness when each approach aligns with its intended support area. To facilitate this process, various technological solutions are identified for each strategy based on their relevance and potential applications. This framework serves as a practical decision-making guide to assist stakeholders in selecting suitable approaches based on their strategic priorities.

The areas of public health priority, as well as research and innovation, were excluded from this analysis, given that existing policies have already been evaluated. Furthermore, any technology integrated to support the fulfillment of these plans inherently drives innovation and, in many cases, requires research to ensure successful implementation.

Virtual reality is a type of technology that enables the creation of synthetic environments where users can engage emotionally, cognitively, and behaviorally, resulting in immersive and meaningful experiences. In the context of dementia care, virtual reality offers several approaches that open new possibilities for early detection and more precise assessment in everyday situations. These include the use of eye-tracking technologies to support diagnostic processes [66], as well as systems that evaluate task performance in daily living [65]. Moreover, virtual reality has been used to train caregivers in the application of care techniques and in responding to behavioral and psychological symptoms [71,72], helping to create more supportive and calming environments. For people living with dementia, it has contributed to maintaining functionality in daily activities and encouraging social interaction [67,70]. It has also proven useful in promoting public understanding and empathy by simulating the sensory and cognitive experiences of a person with dementia [81].

Beyond immersive experiences, another key technological advancement is the use of machine learning algorithms, which offer the potential to identify early cognitive changes through pattern recognition across diverse data sources. Machine learning algorithms are useful tools in dementia research because they can detect subtle and complex patterns across different types of information. In the prediagnostic stage, they are often applied to analyze clinical histories, language use, and physiological indicators. These types of data contribute to identifying early cognitive changes that may not be evident through traditional clinical evaluations [57-59]. Machine learning has also been used to develop scoring instruments such as the Mini-Mental State Examination (MMSE) and the Clock Drawing Test [57-59], supporting the analysis of results and helping to identify individuals who may show signs of cognitive impairment.

Risk reduction is also an area to be addressed. To support these applications, it is necessary to develop longitudinal databases that organize and store diverse sources of information over time. This allows researchers to examine the progression of symptoms and identify traits shared by individuals at higher risk. Several countries have already established national databases with this purpose [63,64].

Smart environments offer a practical solution to provide this data by embedding sensors in the living spaces of people with dementia, enabling passive monitoring of daily activities and health indicators over time. These systems aim to monitor individuals living with dementia and enable them to remain in their homes for a longer period, preserving their autonomy and connection to familiar surroundings and their community. Smart environments can support daily living by tracking routines, providing reminders about medication, hydration, and other activities, and even issuing alerts to caregivers regarding the individual’s condition or location.

As a more accessible and flexible alternative, wearable technologies provide real-time physiological and behavioral data, making them suitable for both urban and rural settings in dementia care. Wearable devices have become valuable tools for caregivers, largely due to their increasing accessibility. In countries such as Japan, these technologies have already been implemented with the primary goals of monitoring vital signs in people living with dementia, preventing wandering, and identifying behavioral patterns over time [82]. To complement this, mobile apps on smartphones and tablets offer a widely accessible means of raising public awareness and supporting early detection through interactive tools.

One of the most accessible ways to reach the general public is through apps available on smartphones and tablets. In some countries, these platforms have been used to raise awareness about the importance of brain health [82], while also providing ready-to-use apps that offer a general overview of an individual’s cognitive status. Closely related to this are efforts to automate existing screening tools. These tools have the potential to expand the reach of detection efforts and support earlier identification of dementia. In addition, several tests have already been adapted into digital formats or modified to deliver fast and simple prediagnostic assessments to a larger segment of the population [67]. Despite their potential, these apps are often limited to predefined functions and lack the ability to adapt dynamically to users’ specific needs.

Generative AI (GenAI) overcomes these limitations by enabling dynamic content creation and real-time conversational interactions, offering new ways to support both people with dementia and their caregivers. The emergence of GenAI has introduced new possibilities through technologies capable of producing text, images, and other multimedia formats using generative models [90]. These models are trained on large datasets to generate content based on learned patterns, with potential applications in dementia care. Their primary use has been to support social interaction and assist in managing the condition [80].

In particular, GenAI has been used to produce text designed to simulate social conversations, support reminiscence therapy, and assist caregivers by providing responses to common questions related to behavioral symptoms and everyday care scenarios. However, the development of these technologies requires advanced technical expertise, meaning that their application will likely come through ready-to-use apps or be driven by researchers and developers working in dementia care.

One particularly promising approach is the use of social robots to provide companionship for people living with dementia. Some of these robots are capable of verbal interaction or offer feedback based on users’ responses. These features make them potentially valuable for supporting emotional well-being and engagement. However, this approach still presents important challenges. The high cost of these devices and the quantity required for use in clinical or institutional settings represent significant financial barriers. In addition, older adults may experience difficulties interacting with such technology, which can lead to resistance or rejection, especially given the digital divide often observed in this age group. Studying user interaction, acceptance, and functionality in local contexts would help identify improvements and inform future development.

Following the global identification of technologies aligned with broad needs, it is necessary to identify the specific strategies and requirements outlined in national or regional dementia plans. Based on these context-specific priorities, relevant technologies can be selected from Table 3. The opportunities for applying these technologies must then be evaluated relative to the social context.

As a next step, current governance frameworks, laws, and regulations governing technology use in these specific environments must be identified. Subsequently, potential limitations and barriers arising from social, cultural, and broader regional characteristics should be determined. Finally, strategic actions can be proposed by synthesizing all contextual factors with available technologies.

The implementation of this framework is demonstrated below using the specific case study of Mexico.

### Mexico as an Illustrative Case Study

Based on the public policies on Mexico analyzed in the previous sections, the identified areas of opportunity can be addressed using specific technologies, as outlined in Table 3.

Mexico, like many other countries, has low rates of dementia diagnosis, with cases often being detected only at advanced stages. Therefore, the development of technologies that enable early prediagnosis represents an essential area of focus. In this context, it is possible to build upon existing elements by integrating digital biomarkers into already established aging surveys, such as MHAS. Combined with the use of AI for pattern detection, this represents a significant opportunity for the development of AI models capable of identifying the main patterns associated with dementia specifically in the Mexican population, while considering the country’s social and cultural context. These models could help inform public policies and support the development of risk indices with high specificity and sensitivity for the Mexican population.

The development of these risk indices could be used as a tool to identify populations with these characteristics or risk factors. In addition, the development of tools based on digital biomarkers, such as voice, gait, or facial expressions, could be used for screening at the primary care level and facilitate referral to specialist physicians. In particular, the creation of databases containing voice recordings from Mexican Spanish speakers is a major unmet need, since currently available databases of voices from people with dementia are predominantly in English, with only a limited number available in Spanish, but not specifically in Mexican Spanish. Consequently, potential biases may arise due to differences in linguistic and cultural characteristics, making localization and adaptation necessary.

MHAS, particularly the Cognitive Aging Ancillary Study (Mex-Cog) substudy, represents a valuable starting point [53]. Expanding the scope of collected data could further enhance its potential. Including additional variables commonly used in international research would strengthen early detection efforts and make it possible to generate risk profiles adapted to the characteristics of the Mexican population. However, the effectiveness of machine learning depends heavily on the availability of comprehensive, high-quality longitudinal data. Furthermore, the integration of digital biomarkers would contribute to the development of more complex and robust databases.

The integration of wearables into the health care system could represent an opportunity for digitalization through the incorporation of devices such as wristbands capable of monitoring vital signs and possible on/off states depending on the condition involved. The aim would be to integrate these data into electronic medical records and facilitate patient monitoring during treatment. These changes should be addressed in the short to medium term, since the health care system is still under development and regulations for the integration of such data into medical records are still required. Furthermore, the integration of digital biomarkers will require restructuring existing surveys, as well as clearly defining which data will be collected, how these data will be harmonized over time, and how they will be anonymized.

As discussed in previous sections, policies should not only focus on diagnosis but also address the context of caregiving. In Mexico, where informal care for older adults is predominantly provided by female family members [91], interventions should prioritize reducing caregiver burden and enhancing the quality of life of older adults. Care-related technologies could be implemented in 3 settings: the home environment, day-care centers, and caregiving systems. Their integration could also be considered the development of smart cities.

The application of technologies within public policies should be considered as part of a comprehensive strategy that also includes digital literacy. In Mexico, this could be promoted through free technology familiarization courses introducing care technologies in day-care centers, as well as through training programs directed toward primary users of these technologies. Furthermore, initiatives for technological familiarization could be delegated to institutions such as Instituto Nacional de las Personas Adultas Mayores (INAPAM), with the objective of improving digital literacy among populations at risk due to age while also promoting the acquisition of new knowledge.

Many caregivers have begun to show interest in implementing technologies in the care process; however, they often do not know how to use them, which represents a significant barrier. Some municipalities in Mexico have already implemented programs that provide wearable GPS devices free of charge to help ensure the safety of people with dementia and prevent them from getting lost [92-94]. Nevertheless, these initiatives have not yet been implemented throughout the entire country. Providing wearable devices that enable monitoring, together with the development of guidelines to support caregivers and disseminating these resources through day-care centers, Sistema Nacional para el Desarrollo Integral de la Familia (DIF) institutions, and health care centers, may represent a viable strategy to facilitate decision-making among caregivers. This approach could also help promote the appropriate use of technologies in ways that benefit both caregivers and people living with dementia.

With the increasing affordability and availability of virtual reality devices, the implementation of these tools in Mexico is becoming more feasible, with potential applications in home care, clinical settings, and day centers. Its implementation in adult day-care centers could be carried out in a phased and controlled manner. This approach would allow for initial user familiarization, enabling subsequent implementation at home during visits from the proposed care system, specifically for rehabilitation purposes. Furthermore, this tool can facilitate personnel training in the early identification of symptoms and the administration of screening tests.

Beyond virtual reality, other technologies could support the care of people with dementia. In the Mexican context, this approach presents both opportunities and challenges. On the one hand, smart home technologies could help address the growing need for home-based care, especially in families where formal caregiving services are limited. On the other hand, the implementation of sensor-based environments may face obstacles such as high installation costs, technical complexity, and concerns about user privacy. These factors highlight the importance of considering culturally and economically appropriate adaptations to ensure the feasibility and acceptance of such technologies in Mexican households.

In fact, studies conducted in Mexico show that WhatsApp (Meta) is one of the most widely used technologies among older adults [95]. Therefore, older adults may be relatively familiar with mobile devices [96]. Consequently, the implementation of applications designed to support cognitive functions and adapted to the local context represents a promising alternative. This idea was reinforced by the Latin American Initiative for Lifestyle Intervention to Prevent Cognitive Decline (LatAm-FINGERS) study, in which cognitive interventions were provided through the use of mobile apps, and participants were instructed on how to use the devices [97].

Although the integration of GenAI into voice assistants represents an emerging area of technological innovation, empirical research regarding user acceptance of these systems in Mexico remains limited. However, existing studies indicate that the main difficulty is the accessibility of these devices, as only 3 of the 40 people interviewed reported having access to one [98]. Nevertheless, their potential for integration into homes and day-care centers was also identified.

This highlights the importance of supporting research and development in the field of health-related technologies. Furthermore, because these models are trained for specific cultural and linguistic contexts, their implementation in Mexico will require careful adaptation to the country’s social and cultural environment.

Despite these limitations, acquiring a single unit for research purposes could offer a valuable opportunity to explore the necessary adaptations for implementation in Mexico. Studying user interaction, acceptance, and functionality in local contexts would help identify improvements and inform future development.

Given these challenges, the above approach offers a more accessible and scalable alternative that may be better suited for immediate integration into dementia care strategies in Mexico.

### Data Management and Regulatory Governance

Although the use and management of data to support tools for treatment and diagnosis, as mentioned in the previous section, represent a significant opportunity for the digitalization of health care in Mexico, there are still no regulations capable of adequately governing these processes. Existing regulations are primarily limited to electronic medical records under NOM-024-SSA3-2012 and NOM-004-SSA3-2012 [99,100]. However, there are currently no specific regulations addressing the integration of wearable devices into these records or processes related to data collection, transfer from applications to clinical servers, and subsequent data use and management.

Furthermore, there are no clinical practice guidelines to support decisions about which devices should be integrated or recommended. This represents a major limitation for the implementation of these technologies in real-world clinical settings in Mexico.

### Limitations and Barriers in Mexico

During the process of implementing technologies in Mexico, several factors arise that complicate their integration. When addressing dementia, the context of older adults cannot be overlooked. In Mexico, the living conditions of older adults vary considerably, resulting in socioeconomic, geographic, and educational differences that create multiple scenarios of difficulty for technology integration.

The integration of technologies often requires infrastructure capable of supporting their operation. For example, voice assistants require constant internet connectivity; however, in many rural areas of Mexico, this is not feasible, thereby creating additional gaps or inequalities in access to health care. Furthermore, the digital divide among older adults reflects differences in their circumstances and access to resources distributed across groups that reflect the limitations of their environment. While many older adults show interest in learning and using technology, they often lack the means to do so. Others may have little or no interest, possibly due to anxiety associated with interacting with technology or because of the lack of infrastructure necessary to sustain its usefulness [95,101,102].

Regarding the possible integration of precision medicine approaches supported by AI, this would require the centralization of data to enable the construction of well-founded individualized analyses. However, although electronic medical records have been mandatory for both public and private institutions in Mexico since 2026, their implementation is still ongoing [103]. In addition, the inclusion of digital biomarkers and data obtained from wearable devices has not yet been clearly defined or regulated.

Another important limitation is the fragmentation of the health care system, which prevents the establishment of a truly universal health care system applicable to the entire population [104]. This creates an additional barrier to the implementation of any public health policy.

### Strategic Directions for Implementation

The integration of technologies into public policies will require a solid foundation for their implementation. At present, for Mexico, the short-term path appears to involve the development of these foundations, through which technologies may later be integrated more smoothly. In the short term, this includes the establishment of adequate infrastructure to guarantee internet access for the entire population, as well as the promotion of digital literacy among older adults both with and without dementia. In addition, caregivers should also be trained through local programs on how to select appropriate devices or technologies according to their specific needs.

Furthermore, in the short term, regulations will also be required to guide health care professionals on how to integrate data obtained from wearable devices into electronic medical records in a precise and regulated manner.

With these foundations in place, and under the assumption of an already established care system, it would be possible to facilitate access for caregivers to basic monitoring devices that support daily life activities and enable integration into electronic medical records.

Once clear regulations regarding data storage and monitoring have been established, the next medium- to long-term step would be to develop dementia risk indices specific to the Mexican population, as well as standardizing metrics and criteria for decision-making based on these data. From this point onward, the pathway would involve the development and implementation of technologies specifically focused on the Mexican context.

## Discussion

The prioritization of dementia as a critical issue has been increasing over time. However, the use of next-generation technologies is not yet fully considered. Therefore, it is necessary to leverage recent technological advances as an opportunity to reactivate the goals established in these international plans. Additionally, it is essential to include measures of the development and application in evaluations of new technologies, such as AI and smart environments.

Since dementia has been declared a global priority and aging has already impacted other countries, many technologies have been developed in those places. These technologies can guide the countries in deciding which paths to follow or avoid, always considering the cultural and social context. Although technology can bring many benefits, barriers such as cost and limited availability reduce its use. To move forward, it is necessary to create strategies that ensure access and promote the use of these tools in dementia care.

Moreover, the implementation of AI in the analysis of medical data can facilitate more accurate diagnoses and early identification of symptoms, which is crucial for appropriate treatment. Telemedicine platforms can also enable remote consultations with specialists, improving accessibility and reducing the burden on the health care system. These advancements can significantly contribute to enhancing the quality of life for individuals living with dementia and optimizing the time and effort of caregivers.

Leveraging these technological advancements requires increased investment in research and innovation. Increased funding for this field would not only improve existing tools but also drive the development of new solutions tailored to the specific needs of each population.

However, ethical issues must also be considered. Some technologies collect personal information, which raises questions about privacy and security. Other tools help people with daily tasks or social activities. In these cases, it is important that the technologies are reliable. There are also concerns about replacing human caregivers with machines, which could reduce the emotional connection in care.

Even if the technologies are available, their success will depend on how well they meet the needs of caregivers, people with dementia, and health care professionals. These tools must be useful, easy to use, and adapted to the reality of the people who need them.

Related to Mexico as a case study, integrating technology into dementia care can not only improve diagnostic and treatment processes but also help reduce geographical and economic barriers that limit access to specialized medical services. Digital tools, such as mobile apps for health monitoring, activity-tracking devices, and case management software, can provide effective solutions for continuous and personalized care, especially in rural areas or communities with limited resources. Although the proposed social programs have significant potential, Mexico’s health system is currently undergoing restructuring, and the effective integration of these technologies will depend on the final performance and organization of the new programs.

## Supplementary material

10.2196/83861 Multimedia Appendix 1 Review of national dementia action plans by country.

## Abbreviations

AD: Alzheimer disease
ADI: Alzheimer’s Disease International
AT: assistive technology
DIF: Sistema Nacional para el Desarrollo Integral de la Familia
ENSANUT: Encuesta Nacional de Salud y Nutrición
FEDMA: Federación Mexicana de Alzheimer
GDO: Global Dementia Observatory
GenAI: generative AI
IMSS: Instituto Mexicano del Seguro Social
INAPAM: Instituto Nacional de las Personas Adultas Mayores
ISSSTE: Instituto de Seguridad y Servicios Sociales de los Trabajadores del Estado
LatAm-FINGERS: Latin American Initiative for Lifestyle Intervention to Prevent Cognitive Decline
Mex-Cog: Cognitive Aging Ancillary Study
MHAS: Mexican Health and Aging Study
MMSE: Mini-Mental State Examination
NGO: nongovernmental organization
PAHO: Pan American Health Organization
SECIHTI: Secretariat of Science, Humanities, Technology, and Innovation
WHO: World Health Organization
