# Supplementary material for: Global Frameworks Toward Effective Integration of Technology in National Dementia Strategies: Narrative Review and Case Study in Mexico
Source: JMIR Aging. 2026 Oct 22;9:e83861. doi: 10.2196/83861 (PMC13717266; doi:10.2196/83861)
Supplement: Supplementary file 1 [file aging-v9-e83861-s001.docx]

**APPENDIX 1 REVIEW COUNTRY ACTION PLANS**

*Summary of Country Action Plans. Author's own creation using data adapted from 44,45,99–102*.

| **Country** | **Name of plan** | **Action Areas** |
| --- | --- | --- |
| Mexico | National Plan for Dementia  (Plan Nacional de Demencia 2024) [47] | 1. Prevention and promotion of mental health.  2. Ensure access to quality services.  3. Timely diagnosis and treatment of individuals affected by the disease.  4. Training of specific and sufficient human resources.  5. Promotion of respect for the human rights of individuals affected by dementia and their caregivers.  6.Increase research on dementia.  7. Periodic evaluation of the effectiveness of proposed actions.  8.Long-term care in the final stages of the disease. |
| Costa Rica | National Plan for Alzheimer’s Disease and Related Dementias Shared Efforts. (Plan Nacional para la enfermedad de Alzheimer y demencias relacionadas esfuerzos compartidos 2014-2024) [50] | 1.Enhance the quality of life for individuals with neurocognitive diseases and those who support them.  2.Motivate individuals with neurodegenerative diseases and their families to choose home as a support environment.  3.Increase support for personal assistants or caregivers.  4.Improve attention and care in long-term care facilities or homes to enhance the quality of life for individuals with neurodegenerative diseases.  5.Develop training programs for healthcare professionals.  6.Promote research.  7.Advocate for general information and social awareness.  8.Emphasize care with a human rights approach.  9.Establish Alzheimer’s disease and related dementias as a national and Central American regional priority. |
| Cuba | Cuban Strategy for Alzheimer's Disease and Dementia Syndromes, "Dementia, a Problem for Everyone" (Estrategia Cubana para la enfermedad de Alzheimer y los sindromes demenciales, "La demencia un problema de todos" 2013) [48] | 1. Plan for improved Care  2. Healthy sector strategy  3. Intersectoral collaboration  4. Early diagnosis  5. Reducing stigma  6. Continuous training for healthcare professionals  7. Research focused on diagnosis  8. Enhancing management and treatment through the establishment of best practices guidelines.  9. Legal instruments for healthcare professionals , individuals living with dementia, and their caregivers. |
| Dominican Republic | Dementia Response Plan in the Dominican Republic  (Plan de respuesta a las demencias en la República Dominicana 2020-2025) [45] | 1. Strengthening public policies for dementia prevention and care.  2. Promoting and preventing dementia to eliminate stigma, improve understanding, and reduce cases.  3. Managing coverage and access to comprehensive care with a focus on prevention, diagnosis, and long-term care.  4. Developing an information system for monitoring, epidemiological surveillance, evaluation, and research to guide decision-making and measure progress. |
| Chile | National Dementia Plan  (Plan Nacional de Demencia 2017) [46] | 1.Prevention of dementias.  2. Diagnosis of dementia at different levels of healthcare. 3.Development of a continuous care system at the local level.  4.Enhancing skills and competencies for caregiving among healthcare professionals and social actors.  5. Monitoring progress.  6. Dissemination of information about dementia to the community.  7.Conducting research focused on aging and dementia.  8. Promoting dementia-friendly communities through the creation of inclusive spaces,the use of technology, and the reduction of stigma.  9. Updating the legal framework related to dementia. |
| Puerto Rico | Action Plan for Puerto Rico  (Plan de Acción para Puerto Rico: Estrategias para mejorar el cuidado y la calidad de vida de las personas con la enfermedad de Alzheimer 2015-2025) [49] | 1.Public health efforts and epidemiological surveillance, 2.Home and community-based care 3.Education and training  4.Diagnosis and treatment 5.Long-term care 6.Financing 7.Public policy. |

References:

45. Plan de respuesta a las demencias en la república dominicana 2020–2025. Ministerio de Salud Pública; 2020. URL: <https://www.alzint.org/u/Dominican-Republic-National-Dementia-Plan.pdf> [Accessed 2026-09-22]

46. Ministerio de Salud de Chile. Plan nacional de demencia 2017. Ministerio de Salud, Gobierno de Chile; 2017. URL: <https://www.minsal.cl/wp-content/uploads/2017/11/PLAN-DE-DEMENCIA.pdf> [Accessed 2026-09-22]

47. García Peña C, Ortega ML, Castro ST, et al. Plan Nacional de Demencia. Instituto Nacional de Geriatría; 2024. ISBN: 978-607-99294-1-1

48. Ministerio de Salud Pública de Cuba. Estrategia Nacional Para El Manejo de La Demencia En Cuba. Ministerio de Salud Pública de Cuba; 2013. URL: <https://www.alzint.org/u/Cuba-National-Dementia-Plan.pdf> [Accessed 2026-10-06]

49. Departamento de Salud. Plan de acción para puerto rico: estrategias para mejorar el cuidado y la calidad de vida de las personas con la enfermedad de Alzheimer. Departamento de Salud, Puerto Rico; 2015. URL: <https://www.alzint.org/u/Puerto-Rico-Plan-de-Accion.pdf> [Accessed 2026-09-22]

50. Plan nacional para la enfermedad de alzheimer y demencias relacionadas esfuerzos compartidos 2014-2024. Consejo Nacional de la Persona Adulta Mayor; 2014. URL: <https://www.alzint.org/u/Costa-Rica-National-Dementia-Plan.pdf> [Accessed 2026-09-22]
